# Supplementary material for: CFD-based design optimization of ducted hydrokinetic turbines
Source: Sci Rep. 2023 Oct 20;13:17968. doi: 10.1038/s41598-023-43724-4 (PMC10589359; doi:10.1038/s41598-023-43724-4)
Supplement: Supplementary file 1 — Supplementary Information. [file 41598_2023_43724_MOESM1_ESM.pdf]

Supplementary Information

A: $k-\omega$ SST turbulence model and automatic wall treatment

The approximation of the Reynolds stress term in Eq. (5) in the main text can be obtained by the Boussinesq hypothesis, which assumes that the Reynolds stress is related to an eddy-viscosity ν_t and the mean velocity gradients, namely (for incompressible flow)

$$-\overline{u_i'u_j'} = \nu_t \left(\frac{\partial \overline{U_i}}{\partial x_j} + \frac{\partial \overline{U_j}}{\partial x_i} \right) - \frac{2}{3} k \delta_{ij} \tag{A.1}$$

where $k = \frac{1}{2}\overline{u_i'u_i'}$ is the turbulent kinetic energy and δ_{ij} is the Kronecker delta function. To obtain k and ν_t in Eq. (A.1), two-equation turbulence models based on $k-\varepsilon$ model[1] or $k-\omega$ model[2] have been developed. Here, ε is the turbulence dissipation rate, and $\omega = \varepsilon/(kC_\mu)$ with $C_\mu = 0.09$ is the so-called specific turbulence dissipation rate. These models include transport equations for k and ε (or ω), and ν_t is obtained as a function of the computed k and ε (or ω) values.

It has been identified by Wilcox[3] that the $k-\varepsilon$ model is robust for regions far from the wall but not accurate when integrating down to the wall (some wall functions are necessary to simulate the viscous sublayer). On the other hand, the $k-\omega$ model offers a more accurate resolution of the viscous sublayer but is less accurate for the far field, e.g., it is over-sensitive to the freestream turbulent conditions[4–6]. On top of both models, the $k-\omega$ based Shear Stress Transport (SST) model[5,7] is proposed, which connects the near-wall region predicted by the $k-\omega$ model and far-field by the $k-\varepsilon$ model using blending functions. In principle, the $k-\omega$ SST model captures the advantage of both models.

In addition to the $k-\omega$ SST model, we apply an automatic wall treatment[8] for near-wall simulation. The automatic wall treatment offers a wall y^+-insensitive simulation, i.e., great flexibility in the first-cell size at the wall. The principle is to set up different wall boundary conditions for k and ω depending on the y^+ value. It has been verified[5,8] that the flow prediction in the near-wall region is insensitive and robust for y^+ from $\mathscr{O}(0.1)$ to $\mathscr{O}(100)$, and the automatic wall treatment has been practically applied for cases with the first cell up to the range in the logarithmic layer, i.e., $y^+ < 300$.

B: Derivation of equations in the rotating reference frame

We start by stating transformations of velocity and acceleration in inertial and rotating reference frames

$$\boldsymbol{U} = \boldsymbol{U}_R + \boldsymbol{\Omega} \times \boldsymbol{r} \tag{B.1}$$

$$\frac{d\boldsymbol{U}}{dt} = \left[\frac{d\boldsymbol{U}}{dt}\right]_R + \frac{d\boldsymbol{\Omega}}{dt} \times \boldsymbol{r} + 2\boldsymbol{\Omega} \times \boldsymbol{U}_R + \boldsymbol{\Omega} \times \boldsymbol{\Omega} \times \boldsymbol{r}, \tag{B.2}$$

where variables with subscript R are evaluated in the rotating reference frame. Here we consider the case $d\boldsymbol{\Omega}/dt = 0$ that is consistent with our application. Derivations of Eq. (B.2) can be found in standard textbooks on dynamics[9].

Substituting Eq. (B.1) into Eq. (4a) and making use of the fact that divergence of a curl of a vector is zero, we obtain the continuity equation in rotating reference frame $\nabla \cdot \boldsymbol{U}_R = 0$. In order to derive momentum equations in the rotating reference frame, we consider Eq. (4b) with material derivatives replacing the unsteady and convection terms, to which we can then substitute Eq. (B.2) to obtain

$$\left[\frac{d\boldsymbol{U}}{dt}\right]_R + 2\boldsymbol{\Omega} \times \boldsymbol{U}_R + \boldsymbol{\Omega} \times \boldsymbol{\Omega} \times \boldsymbol{r} = -\frac{1}{\rho}\nabla p + \nabla \cdot (\nu \nabla \boldsymbol{U}) \tag{B.3}$$

The viscous term $\nabla \cdot (\nu \nabla \boldsymbol{U})$ can be transformed to $\nabla \cdot (\nu \nabla \boldsymbol{U}_R)$ using the fact that $\nabla \cdot [\nu \nabla (\boldsymbol{\Omega} \times \boldsymbol{r})] = 0$. We then expand the material derivative in Eq. (B.3) to obtain the momentum equations in the rotating reference frame:

$$\frac{\partial \boldsymbol{U}_R}{dt} + \nabla \cdot (\boldsymbol{U}_R \boldsymbol{U}_R) = -\frac{1}{\rho}\nabla p + \nabla \cdot (\nu \nabla \boldsymbol{U}_R) - 2\boldsymbol{\Omega} \times \boldsymbol{U}_R - \boldsymbol{\Omega} \times \boldsymbol{\Omega} \times \boldsymbol{r} \tag{B.4}$$

While Eq. (B.4) can be considered the final equation in a rotating reference frame, it can be simplified further for easier implementation in the finite volume method. For this purpose, we re-arrange the convection term as

$$\begin{aligned} \nabla \cdot (\boldsymbol{U}_R \boldsymbol{U}_R) &= \nabla \cdot [\boldsymbol{U}_R(\boldsymbol{U} - \boldsymbol{\Omega} \times \boldsymbol{r})] \\ &= \nabla \cdot (\boldsymbol{U}_R \boldsymbol{U}) - \nabla \cdot \boldsymbol{U}_R(\boldsymbol{\Omega} \times \boldsymbol{r}) - \boldsymbol{U}_R \cdot \nabla(\boldsymbol{\Omega} \times \boldsymbol{r}) \\ &= \nabla \cdot (\boldsymbol{U}_R \boldsymbol{U}) - \boldsymbol{\Omega} \times \boldsymbol{U}_R \end{aligned} \tag{B.5}$$

where the following equality is used for the third line

$$\begin{aligned}
\boldsymbol{U}_R \cdot \nabla(\boldsymbol{\Omega} \times \boldsymbol{r}) &= U_{R_l} \frac{\partial}{\partial x_l} \varepsilon_{ijk} \Omega_j r_k \\
&= U_{R_l} \varepsilon_{ijk} \Omega_j \frac{\partial r_k}{\partial x_l} \\
&= U_{R_l} \varepsilon_{ijk} \Omega_j \delta_{kl} \\
&= \varepsilon_{ijl} \Omega_j U_{R_l} \\
&= \boldsymbol{\Omega} \times \boldsymbol{U}_R.
\end{aligned} \tag{B.6}$$

The final equations in the rotating reference frame therefore yield

$$\begin{cases} \nabla \cdot \boldsymbol{U} = 0 \\ \frac{\partial \boldsymbol{U}_R}{\partial t} + \nabla \cdot (\boldsymbol{U}_R \boldsymbol{U}) = -\frac{1}{\rho} \nabla p + \nabla \cdot (\nu \nabla \boldsymbol{U}) - \boldsymbol{\Omega} \times \boldsymbol{U}, \end{cases} \tag{B.7}$$

which is Eq. (7) in the main paper. For a steady solution of Eq. (B.7), we can consider $\boldsymbol{U}$ as the unknowns that are consistent with the outer stationary region and use one unified code for the solution with some minor modifications (in terms of forcing $\boldsymbol{\Omega} \times \boldsymbol{U}$ and the $\boldsymbol{U}_R$ term leading to a correction in computing the cell face flux) for the rotating region.

C: Data Transfer at the Interface in the Rotating-Sliding Mesh

The interfaces involved in the turbine problem are shown in Figure 7 in the main paper. For cells on each side of the interface, the flux value on the cell face toward the interface needs to be constructed. This cannot be obtained from a standard interpolation method because the cells on two sides of the interface are non-conformal, i.e., with non-overlapping cell faces from the two sides. Given the donor and target meshes at the interface, our goal is to compute the flux value at the cell face of the target mesh. This requires reconstruction of the cells (as well as cell-centered properties) on the donor side into a "supermesh"[10] whose faces (at the interface) contain all nodes of the target mesh, so that the cell face flux on the target side can be computed by interpolation between the supermesh and target mesh.

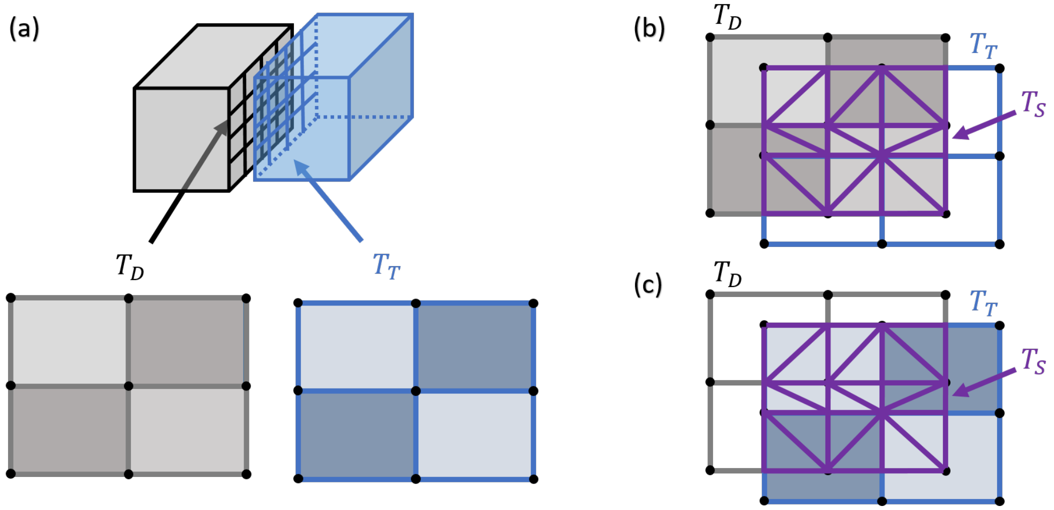

Figure S1. An example of supermesh construction at a non-conformal interface. (a) Donor mesh T_D and target mesh T_T are non-conformal. A triangular supermesh T_S with the shaded area denoting elements (b) on T_D and (c) on T_T.

In general, the interpolation via supermesh involves a Galerkin projection method described in detail in Farrell and Maddison[11]. Here we provide a simple example to illustrate the gist of the approach, as sketched in Figure S1. With donor mesh T_D and target mesh T_T that are non-conformal (Figure S1a), our goal is to construct the supermesh T_S that serves as a common ground for interpolation. The supermesh T_S has to satisfy the properties:

1. Nodes on T_S contains all the nodes on T_D and T_T (and intersections of their edges).

2. For every (face cell) element in T_S, the intersection of it with any element of T_D or T_T must either be zero or the whole element.

The connection between T_D and T_S is shown in Figure S1b, with the shaded area denoting cell faces of T_D. Since each cell face of T_D is now perfectly split into triangle cell faces of T_S, we can assign properties to the center of the volume supermesh (associated with triangle faces) according to the area ratio of the triangles. In other words, the property at T_D is distributed into fractions according to the area fraction of triangles in T_S. The properties at T_S can then be used for interpolation with that at the target cell T_T since each target cell face is also perfectly split into triangle faces of T_S (Figure S1c). Specifically, we interpolate between the volume cell of T_S and the corresponding volume cell at the T_T side to obtain the flux value (which is associated with the corresponding triangle area). These fluxes are then added to form the value for a single cell face of T_T.

D: Analytical connection between FFD points and enclosed geometry

In this section, we explain how to obtain the analytical connection of FFD points to the enclosed geometry. While the FFD method is versatile in handling geometries in different dimensions, we focus on an application consistent with our work, which involves the FFD points creating a lattice box in $\mathbb{R}^3$ that encloses a three-dimensional (3D) geometry within the box. Essentially, the analytical connection is to establish a mapping $\mathbb{R}^3 \to \mathbb{R}^3$ for each point inside the box. The mapping can be established by making use of the tri-variate B-spline function:

$$X(u,v,w) = \sum_{i=0}^{N_u-1} \sum_{j=0}^{N_v-1} \sum_{k=0}^{N_w-1} B_{i,m_u}(u) B_{j,m_v}(v) B_{k,m_w}(w) P_{i,j,k}, \tag{D.1}$$

where $X(u,v,w) \in \mathbb{R}^3$ is the coordinate of the volume enclosed by FFD points (and our geometry of interest is a part of this volume), parameterized by u, v, and w (all $\in [0,1]$). The indexes i, j, and k loop in three directions of the lattice in $\mathbb{R}^3$, N_u, N_v, and N_w are the number of FFD points in each direction of the lattice, and $P_{i,j,k} \in \mathbb{R}^3$ are coordinates of the lattice FFD points. The indexes, m_u, m_v and m_w are prescribed degrees of the B-spline basis functions in three directions. The B-spline basis function is defined recursively by (taking the i-direction as an example)

$$B_{i,0}(x) = \begin{cases} 1 & \text{if} \quad t_i \le x < t_{i+1} \\ 0 & \text{otherwise} \end{cases}, \tag{D.2}$$

$$B_{i,k}(x) = \frac{x - t_i}{t_{i+k} - t_i} B_{i,k-1}(x) + \frac{t_{i+k+1} - x}{t_{i+k+1} - t_{i+1}} B_{i+1,k-1}(x), \tag{D.3}$$

where t_i is the so-called open knot vector that is determined by choice of N_u and m_u [12,13].

With Eq. (D.1), the sensitivity of the geometry to a particular FFD point, in terms of derivative, can be analytically expressed as

$$\frac{\partial X(u,v,w)}{\partial P_{i,j,k}} = B_{i,m_u}(u) B_{j,m_v}(v) B_{k,m_w}(w). \tag{D.4}$$

Since $X \in \mathbb{R}^3$ and $P \in \mathbb{R}^3$, the derivative on the LHS of Eq. (D.4) requires further clarification. It essentially means that the three piecewise derivatives of one $\mathbb{R}^3$ vector with respect to another $\mathbb{R}^3$ vector are equal with one another and also equal to the RHS.

In our case, linked FFD points are used as one degree of freedom (DoF). The derivative of the geometry coordinates with respect to the particular DoF motion can be computed by summing the contribution of each FFD point using Eq. (D.4).

E: Partial Derivative Computation in the Adjoint Method

This section presents the underlying principle of how the partial derivatives in the adjoint method can be computed. All these derivatives are computed by backward automatic differentiation[14] in DAFoam with graph coloring method for acceleration[15].

Four types of partial derivatives are involved:

$$\underbrace{\frac{\partial C_P}{\partial \boldsymbol{s}}}_{1\times M}, \underbrace{\frac{\partial C_P}{\partial \boldsymbol{x}}}_{1\times N_x}, \underbrace{\frac{\partial \boldsymbol{R}}{\partial \boldsymbol{s}}}_{M\times M}, \underbrace{\frac{\partial \boldsymbol{R}}{\partial \boldsymbol{x}}}_{M\times N_x}. \tag{E.1}$$

For the former two, we consider $P = \Omega \int \boldsymbol{r} \times p d\mathscr{S}$, where p is the pressure, $\boldsymbol{r}$ is the distance to the rotating axis, the integration is over the blade surface with differential element $d\mathscr{S}$. It is clear that C_P depends on both the state variable $\boldsymbol{s}$ (i.e., pressure p) and design variable $\boldsymbol{x}$ (i.e., the integration surface). The derivative $\partial C_P / \partial \boldsymbol{s}$ can be simply computed by perturbing the pressure field in the flow solution. For computing $\partial C_P / \partial \boldsymbol{x}$, we write $P = \Omega \sum_i \boldsymbol{r}_i \times p_i \Delta\mathscr{S}_i$ which is in discrete form. A perturbation in $\boldsymbol{x}$ results in perturbations of the FFD points, which deforms the geometry. This, in turn, leads to the surface mesh deformation that affects the $\boldsymbol{r}_i$ and $\Delta\mathscr{S}_i$. The partial derivative $\partial C_P / \partial \boldsymbol{x}$ can therefore be computed using the chain rule to connect all processes.

For the latter two, we consider the discretized governing equation in residual form, i.e., $\boldsymbol{R} = 0$. Perturbation of $\boldsymbol{s}$ changes the velocity field, pressure field, and the velocity flux (at cell faces) so that $\boldsymbol{R}$ is perturbed. The derivative $\partial \boldsymbol{R}/\partial \boldsymbol{s}$ can then be computed correspondingly. Perturbation of $\boldsymbol{x}$ leads to the deformation of all computational mesh in the full fluid domain. The construction of velocity flux (or more precisely, the coefficients in the algebraic governing equation) is thus affected, leading to the computation of $\partial \boldsymbol{R}/\partial \boldsymbol{x}$ correspondingly.

F: Discrete grid-search in a lower-dimensional design space

This section describes our effort in a brute-force grid search of a lower-dimensional design space. Taking the baseline duct design as a reference[16], we first create 5 sets of duct configurations, each set representing one type of duct with 5 varying designs each. As sketched in Figure S2, the 5 sets are: (1) varying outlet area from the baseline design; (2) varying outlet area from the baseline design with throat section as the duct inlet; (3) varying both inlet and outlet areas from the baseline design with a finite-thickness duct (outer surface simply as a straight line); (4) varying outlet area from the baseline design with a flange (inspired by Ohya and Karasudani[17]); (5) varying inlet area from the baseline design. In total, these represent 25 duct designs, with the ratio of maximum and minimum duct areas ranging from 1.25 to 2.75. For each of these duct designs, other design variables considered are listed below with discrete values:

- λ: 3, 3.5, 4, 4.5, 5, 5.5, 6, 6.5, 7, 7.5
- Blade root pitch: 10°, 20°, 30°, 40°, 45°
- Twist profiles: Baseline blade designs A and B
- Tip gap ratio: $0.09R$, $0.2R$

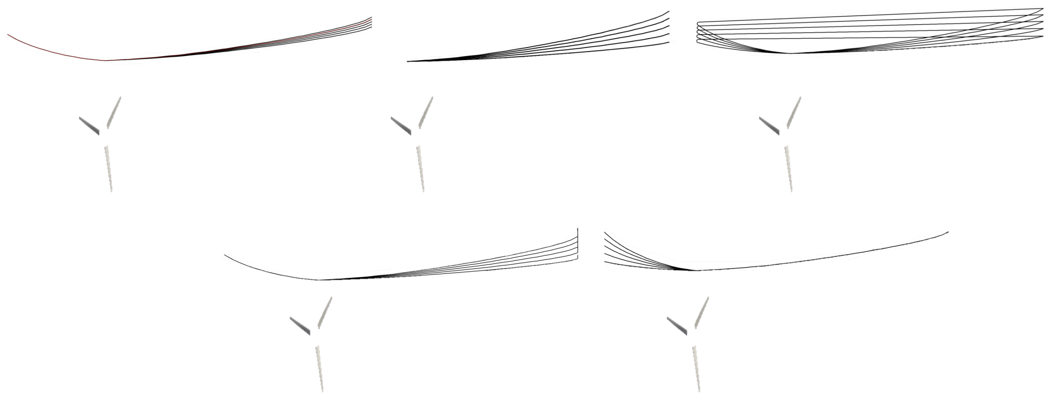

Figure S2. Five sets of duct designs in the grid-search study.

The RANS-MRF solver is used for each design to evaluate its performance on a coarse grid with 1-2 million cells (even coarser than M0). In total, about 450 cases are run with results of their efficiency C_P shown in Figure S3. Since it is difficult to visualize results in the design space of more than three dimensions, we simply plot all results as functions of λ, i.e., for each λ, a large number of C_p values resulting from varying other design variables are stacked. While it is not our goal to further distinguish designs at each λ, it is clear that the maximum C_p among all $\mathcal{O}(450)$ cases is only 38% evaluated by the RANS-MRF. We pick up a few designs with relatively high C_p and re-evaluate their performances using the higher-fidelity URANS solver on the M0 grid, with results also shown in Figure S3. We see that the maximum efficiency computed by the URANS is 45%, which happens to correspond to our baseline design B (in terms of both the duct and blade geometry) but is still lower than 46% from the unducted Bahaj model. Therefore, a simple brute-force grid search, as performed here, does not provide any duct turbine design with higher efficiency than that of standard unducted turbines.

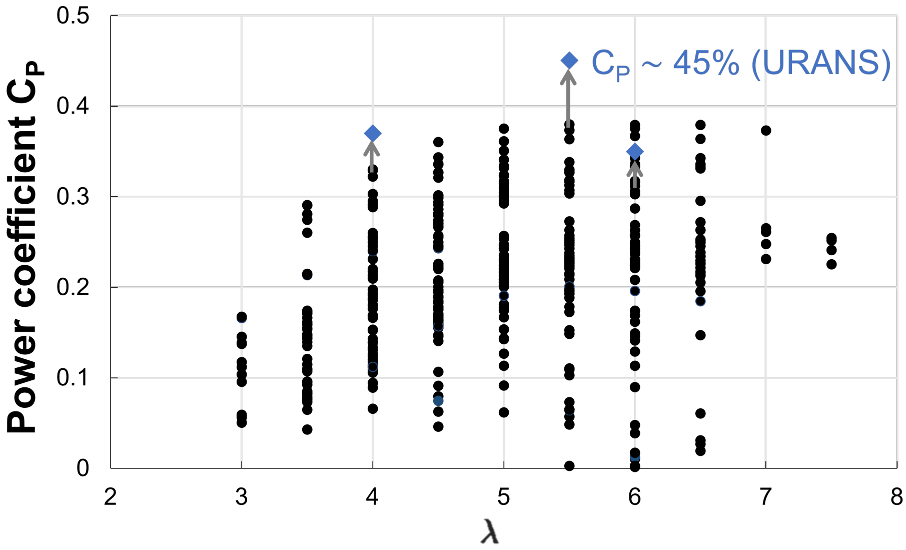

Figure S3. C_P's obtained from $\mathcal{O}(450)$ the RANS-MRF simulations for discrete points in the design space, with results stacked to each λ. The URANS re-evaluations of some designs with relatively high C_P's from the RANS-MRF yield results shown by diamonds.

References

1. Jones, W. P. & Launder, B. E. The prediction of laminarization with a two-equation model of turbulence. *Int. journal heat mass transfer* **15**, 301–314, DOI: 10.1016/0017-9310(72)90076-2 (1972).
2. Wilcox, D. C. Reassessment of the scale-determining equation for advanced turbulence models. *AIAA journal* **26**, 1299–1310, DOI: 10.2514/3.10041 (1988).
3. Wilcox, D. C. Comparison of two-equation turbulence models for boundary layers with pressure gradient. *AIAA journal* **31**, 1414–1421, DOI: 10.2514/3.11790 (1993).
4. Kok, J. C. Resolving the dependence on freestream values for the k-turbulence model. *AIAA journal* **38**, 1292–1295, DOI: 10.2514/2.1101 (2000).
5. Menter, F. R., Kuntz, M. & Langtry, R. Ten years of industrial experience with the sst turbulence model. *Turbul. heat mass transfer* **4**, 625–632 (2003).
6. Menter, F. R. Review of the shear-stress transport turbulence model experience from an industrial perspective. *Int. journal computational fluid dynamics* **23**, 305–316, DOI: 10.1080/10618560902773387 (2009).
7. Menter, F. R. Two-equation eddy-viscosity turbulence models for engineering applications. *AIAA journal* **32**, 1598–1605, DOI: 10.2514/3.12149 (1994).
8. Kalitzin, G., Medic, G., Iaccarino, G. & Durbin, P. Near-wall behavior of rans turbulence models and implications for wall functions. *J. Comput. Phys.* **204**, 265–291, DOI: 10.1016/j.jcp.2004.10.018 (2005).
9. Taylor, J. R. & Taylor, J. R. *Classical mechanics*, vol. 1 (Springer, 2005).
10. Farrell, P. E., Piggott, M. D., Pain, C. C., Gorman, G. J. & Wilson, C. R. Conservative interpolation between unstructured meshes via supermesh construction. *Comput. methods applied mechanics engineering* **198**, 2632–2642, DOI: 10.1016/j.cma.2009.03.004 (2009).
11. Farrell, P. & Maddison, J. Conservative interpolation between volume meshes by local galerkin projection. *Comput. Methods Appl. Mech. Eng.* **200**, 89–100, DOI: 10.1016/j.cma.2010.07.015 (2011).
12. Kenway, G., Kennedy, G. & Martins, J. R. A cad-free approach to high-fidelity aerostructural optimization. In *13th AIAA/ISSMO multidisciplinary analysis optimization conference*, 9231, DOI: 10.2514/6.2010-9231 (2010).
13. Reid, J. & He, P. Free-form deformation (ffd) (2021).
14. Martins, J. R. & Ning, A. *Engineering design optimization* (Cambridge University Press, 2021).

15. Gebremedhin, A. H., Manne, F. & Pothen, A. What color is your jacobian? graph coloring for computing derivatives. *SIAM review* **47**, 629–705, DOI: 10.1137/S0036144504444711 (2005).

16. Knight, B., Freda, R., Young, Y. L. & Maki, K. Coupling numerical methods and analytical models for ducted turbines to evaluate designs. *J. Mar. Sci. Eng.* **6**, DOI: 10.3390/jmse6020043 (2018).

17. Ohya, Y. & Karasudani, T. A shrouded wind turbine generating high output power with wind-lens technology. *Energies* **3**, 634–649, DOI: 10.3390/en3040634 (2010).
